# Ex vivo mRNA expression of toll-like receptors during latent tuberculosis infection

**DOI:** 10.1186/s12865-021-00400-4

**Published:** 2021-01-28

**Authors:** Birhan Alemnew, Soren T. Hoff, Tamrat Abebe, Markos Abebe, Abraham Aseffa, Rawleigh Howe, Liya Wassie

**Affiliations:** 1 College of Health Sciences, Department of Medical Laboratory Sciences, Woldia University, Woldia, Ethiopia; 2 Armauer Hansen Research Institute, Addis Ababa, Ethiopia; 3 Statens Serum Institute, Copenhagen, Denmark; 4 Department of Microbiology, Immunology and Parasitology, College of Health Sciences, School of Medicine, Addis Ababa University, Addis Ababa, Ethiopia

**Keywords:** TLR, mRNA, Latency, Tuberculosis, Children

## Abstract

**Background:**

Understanding immune mechanisms, particularly the role of innate immune markers during latent TB infection remains elusive. The main objective of this study was to evaluate mRNA gene expression patterns of toll-like receptors (TLRs) as correlates of immunity during latent TB infection and further infer their roles as potential diagnostic biomarkers.

**Methods:**

Messenger RNA (mRNA) levels were analysed in a total of 64 samples collected from apparently healthy children and adolescents latently infected with tuberculosis (*n* = 32) or non-infected (*n* = 32). Relative expression in peripheral blood of selected genes encoding TLRs (TLR-1, TLR-2, TLR-4, TLR-6 and TLR-9) was determined with a quantitative real-time polymerase chain reaction (*qRT-PCR*) using specific primers and florescent labelled probes and a comparative threshold cycle method to define fold change. Data were analysed using Graph-Pad Prism 7.01 for Windows and a *p*-value less than 0.05 was considered statistically significant.

**Results:**

An increased mean fold change in the relative expression of TLR-2 and TLR-6 mRNA was observed in LTBI groups relative to non-LTBI groups (*p* < 0.05), whereas a slight fold decrease was observed for TLR-1 gene.

**Conclusions:**

An increased mRNA expression of TLR-2 and TLR-6 was observed in latently infected individuals relative to those non-infected, possibly indicating the roles these biomarkers play in sustenance of the steady state interaction between the dormant TB bacilli and host immunity.

**Supplementary Information:**

The online version contains supplementary material available at 10.1186/s12865-021-00400-4.

## Introduction

*Mycobacterium tuberculosis* (*M. tuberculosis*), a causative agent of tuberculosis, is responsible for death of nearly 1.2 million HIV-negative individuals globally [1]. Understanding the immune mechanism during latency, a state of persistent immune response to stimulation by *M. tuberculosis* antigens [2–4], has long been elusive for decades, while the global estimate shows just under a quarter (~ 23%) of the global population is labelled as latently infected [5]. The host innate immune response is the first line of defence against invading pathogens and is vital for the initial defence against *M. tuberculosis* and activation of the adaptive immune response [6]. Toll-like receptors (TLRs) are a class of proteins that are single, membrane-spanning receptors mostly expressed on leukocytes, including macrophages that have significant role in TB pathogenesis, one of the antigen-processing cells and having a role during granuloma formation [7]. These receptors generally recognize structurally conserved molecules derived from microbes such as bacteria and viruses [8, 9] and mediate cell activation, leading to induction of pro inflammatory cytokines, dendritic cell maturation and apoptosis [8, 10]. Due to lack of long lasting immunity, little attention has been given to understanding the role of innate immunity in protection against latent TB [11]; however, this notion has now been changing with recent concepts of “trained immunity”, where some of the main features and traits of the adaptive immunity, such as specificity and memory, are also shared by innate markers [12]. Identification of host biomarkers has long been a way forward in understanding the immunology of latent TB [6] and contributing to development of these tools is of immense importance. To this effect, we aimed to evaluate the expression of selected TLR mRNAs in peripheral blood as correlates of immunity during latent TB infection.

## Methods

### Study setting

Using purposive sampling, a total of 64 apparently healthy children and adolescents, aged between 11 and 18 years, and who have been recruited from different schools in Addis Ababa, capital city of Ethiopia (between 2014 and 2016), a country that has been reported as one of the highest TB burden settings globally [1], were included in this study. Children below the age of 11 were excluded for the sake of ethical regulations in Ethiopia, restricting blood volumes needed to conduct the proposed research. Participants had no known illness and were asymptomatic upon screening during enrolment and were categorized as latently infected with TB (*N* = 32), after being tested positive by tuberculin skin testing (TST) or quantiferon (QFT) and non-infected (*N* = 32) after being tested negative by TST or QFT as previously described [13]. An indurations size of 10 mm and above was considered as a cut-off to define tuberculin reactivity [14]. Similarly, an IFN-γ level above 0.35 International Units (IU)/mL for (TB Antigen-Nil) was considered as positive, while testing using QFT, according to the manufacturer’s instruction. Participants had no known history of contact with a known TB patient or TB disease, with the majority having history of BCG vaccination and no concomitant infections (such as parasites) (Table 1). Diagnosis of stool helminthic parasites/ova was done using the Kato-Katz technique as described in our previous study [13]. Participants who were HIV positive or those with a history of allergy or chronic infection at the time of clinical screening were excluded from the study.

**Table 1:** Socio-demographic and clinical characteristics of study participants (*N* = 64)

Variables	Overall N (%)	LTBI group N (%)	Non-LTBI group N (%)	***P***-value
Sex				0.617
Male	32 (50)	17 (53.1)	15 (46.9)	
Female	32 (50)	15 (46.9)	17 (53.2)	
Age (Years)				0.802
11–14	32 (50)	16 (50.0)	16 (50.0)	
15–18	32 (50)	16 (50.0)	16 (50.0)	
BMI (Kg/M^2^)^a^				0.230
Underweight (< 18.5)	33 (51.56)	18 (56.2)	20 (62.5)	
Normal (18.5–24.9)	29 (45.3)	12 (37.5)	12 (37.5)	
Overweight (> 24.9)	2 (0.03)	2 (0.6)	0 (0.0)	
BCG scar				0.118
Present	33 (51.6)	13 (40.6)	20 (62.5)	
Absent	25 (39.1)	15 (46.9)	10 (31.2)	
Indeterminate	3 (4.7)	1 (3.1)	2 (6.3)	
Not recorded	3 (4.7)	3 (9.4)	0 (0.0)	
Parasitic infections				0.026
Yes	18 (28.1)	13 (40.6)	5 (15.6)	
No	46 (71.9)	19 (59.4)	27 (84.4)	

^a^*BMI* body mass index and calculations were based on the Ethiopian demographic and health survey [15]

### Analysis of mRNA expression using quantitative real-time PCR (*qRT-PCR*)

Approximately 2.5 ml of venous blood was collected into PAXgene Blood RNA Kit (PreAnalytiX, QIAGEN); reverse transcribed into complementary DNA (cDNA) using Omniscript Reverse Transcription Kit (QIAGEN, Germany) and oligo (dT) primers (Promega, USA) as described previously [13] and stored at -20 °C until assayed further by quantitative real-time PCR (*qRT-PCR*). To analyze the relative mRNA expression of selected TLRs in whole blood, *qRT-PCR* was performed using QuantiTect Reverse Transcription PCR Kit (QIAGNE, Germany). The amount and purity of all cDNA samples were measured using NanoDrop 2000 Spectrophotometer (Thermo Scientific, Amersham Biosciences, UK), adjusted and normalized to a final concentration of 0.2 μg/μl and a total of 1 μg of cDNA was used as a starting template in a total reaction volume of 12.5 μl. Specific primers and florescent-labelled probes (Eurofins MWG Operon) (Table 2) were used at a final concentration of 0.5 μM and 0.3 μM, respectively to span exon-intron junctions to prevent amplification of genomic DNA and amplicons of fewer than 150 base pairs to enhance the efficiency of PCR amplification. All the PCR mixtures were prepared using Corbett Robotics (Corbett Research, Australia) and run in duplicates including non-template controls using Rotor-Gene (RG-3000) thermo-cycler (Corbett Research, Australia). The *qRT-PCR* cycling was carried out under the following conditions: initial Taq enzyme heat activation step at 95 °C for 15 min, followed by 40 repeats of three step cycling, denaturation step 94 °C for 10 s, primer/probe annealing step ranging between 58 °C and 60 °C for 20 s and final extension/polymerization step at 72 °C for 30 s. In all experiments, data were acquired at extension phase (on FAM/Sybr channel) and analyzed using Rotor-Gene Real-Time Analysis Software 6.0 (Corbett Research, Australia). Human ribosomal protein (HuPO) was used as a housekeeping gene and internal control and a non-template tube as a negative control throughout the experiment; yielding a PCR efficiency of 97% (See Supplementary data). The threshold cycle (CT), the PCR cycle at which the fluorescent signal of the reporter dye crosses an arbitrarily point, was set at the exponential phase of amplification and used as the quantitative endpoint of *qRT-PCR* as described earlier [16]. Comparative CT method (also known as 2^(−ΔΔCT)^ was used to describe the fold change in the relative expression of TLR genes as previously described [16]. Briefly, the non-LTBI group was considered as calibrator and the LTBI as target group to estimate the change in the CT values (ΔCT); all CT values were initially normalized to the CT values of HuPO. The computed numbers indicate the fold change in the expression of a target gene in latently infected group relative to non-infected. A similar analysis was performed to compare the relative expression of the genes across age, where children under 15 years were considered as calibrator groups and adolescents (those 15 years and above) as target groups. Furthermore, to appreciate the relative expression of TLRs localized to the cell surface (TLR-1, TLR-2, TLR-4 and TLR-6) to enodsomal (TLR-9) regions, we compared the ratio of comparative CT values of TLRs in TST positive and negative groups. This work was conducted at the Armauer Hansen Research Institute (AHRI) laboratory.

**Table 2:** Primers and probes sequences (5′-3′) used in this study

Primers and probes	Sequence (5′-3′)
HuPO Primer (5′-3′)	forward: GCT TCC TGG AGG GTG TCC
	reverse: GGA CTC GTT TGT ACC CGT TG
Probe (5′-6-FAM---TAMRA-3′):	TGC CAG TGT CTG TCT GCA GAT TGG
TLR-1 Primer (5′-3′)	forward: CAG TGT CTG GTA CAC GCA TGG
	reverse: TTT CAA AAA CCG TGT CTG TTA AGA G
Probe (5′-6-FAM---TAMRA-3′):	TGC CCA TCC AAA ATT AGC CCG TTC C
TLR-2 Primer (5′-3′)	forward: GGC CAG CAA ATT ACC TGT GTG
	reverse: AGG CGG ACA TCC TGA ACC T
Probe (5′-6-FAM---TAMRA-3′):	TCC ATC CCA TGT GCG TGG CC
TLR-4 Primer (5′-3′)	forward: GAG CCT TTT CTG GAC TAT CAA G
	reverse: TCC AAT GGG GAA GTT CTC TAG
Probe (5′-6-FAM---TAMRA-3′):	AGA TTT GTC TCC ACA GCC ACC AGC
TLR-6 Primer (5′-3′)	forward: GAA GAA GAA CAA CCC TTT AGG ATA GC
	reverse: AGG CAA ACA AAA TGG AAG CTT
Probe (5′-6-FAM---TAMRA-3′):	TGC AAC ATC ATG ACC AAA GAC AAA GAA CCT
TLR-9 Primer (5′-3′)	forward: GGA CC + T CTG GTA CTG CTT CCA
	reverse: AAG CTC GTT GTA CAC CCA GTC T
Probe (5′-6-FAM---TAMRA-3′)	ACG ATG CCT TCG TGG TCT TCG ACA AA

*HuPO* human acidic ribosomal protein, *TLR* toll-like receptor, *FAM* 6-carboxyfluorescein, *TAMRA* tetramethylrhodamine

### Data analyses

Data from *qRT-PCR* (2^(−ΔΔCT)^) were exported to GraphPad Prism Version 7.01 for Windows (GraphPad Software, La Jolla California USA, www.graphpad.com) and SPSS Version 20.0 for further statistical analyses. Categorical variables were presented as counts and percentages and compared using Chi square test. For two groups, analyses of the fold change in the relative expression of the TLRs were done using non-parametric ANOVA (Kruskal-Wallis test with Dunn’s multiple comparison tests). In addition, Spearman linear correlation (r) was done to compare the correlation between the surface and intracellular TLRs. In all instances, a *P*-value < 0.05 was considered statistically significant.

## Results

### Relative mRNA expressions of TLRs in LTBI and non-LTBI groups

The relative mRNA expressions of TLRs (TLR-1, TLR-2, TLR-4, TLR-6, and TLR-9), normalized to HuPO, were compared between latently infected and non-infected individuals. As the numerical value of the CT inversely relates to the amount of amplicon in the reaction [16], threshold values above 30 were considered as weak fluorescent signals. Results are presented as fold change of target gene expression in a target sample relative to a reference sample, normalized to a reference gene. With the relative gene expression set to 1 for the reference sample, the ΔΔ*CT* is equal to 0, resulting in 2^0^, i.e., 1 [17], and thus the mean fold change greater than one was considered as up-regulation or increased expression of a particular gene.

**Fig. 1:**
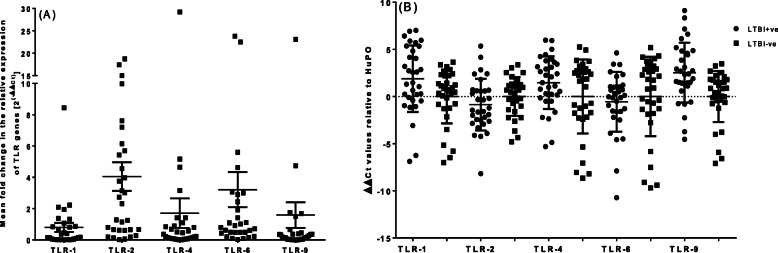
Mean fold change in the mRNA expression of TLR in LTBI individuals relative to those non-infected; error bars indicate mean with standard error of the mean (SEM); TLR = toll-like receptor. Figure on the left side **a** shows the comparison with the outcome of LTBI, each LTBI compared with the average non-LTBI group (ratio). Figure on the right side **b** shows the same comparison (compares LTBI vs. Non-LTBI groups) with the outcome TLR index (normalized to a housekeeping gene, HuPO)

An increased mean fold change in the mRNA expression of TLRs was observed in latently infected individuals relative to those non-infected (Fig. 1). This increase was four-fold for TLR-2 gene (mean with SEM: 4.04, 0.91 and 95% CI (2.18, 5.91)) and three-fold for TLR-6 (mean with SEM: 3.21, 1.12 and 95% CI (0.93, 5.50)) and approaching a two-fold increase for TLR-4 and TLR-9 (mean with SEM: 1.71 ± 0.95 and 95% CI (0.2, 3.7) and 1.59 ± 0.8 and 95% CI (0.07, 3.3)), respectively (*p* = 0.0001). On the other hand, a slight decrease in the mean fold change in the mRNA expression of TLR-1 was observed in those latently infected compared to non-infected (mean with SEM: 0.80, 0.29, and 95% CI (0.20, 1.39)) (Fig. 1a). The difference looks more apparent when the normalized ΔΔCt values are compared between LTBI infected and those non-infected (Fig. 1b).

### Relative mRNA expressions of TLRs among adolescents and children

**Fig. 2:**
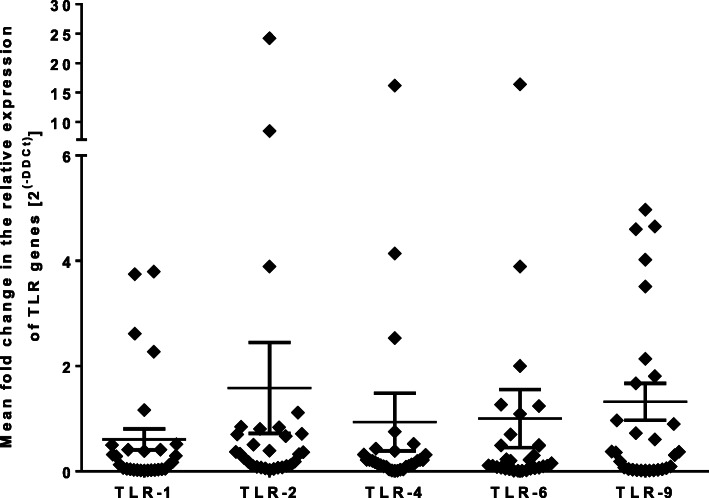
Mean fold change in mRNA expression of TLRs among adolescents relative to children; error bars indicate mean values with standard error of the mean (SEM); TLR = toll-like receptor

To understand whether adolescents have distinct mRNA expression patterns compared to younger children, the mean fold change in the expression of TLRs was also compared across age, where those aged between 11 and 14 years were considered as children and those above 15 years as adolescents and no significant difference was observed between the two groups (Data not shown). Similarly, no apparent difference was noted between male and female participants (*P* > 0.05) (Fig. 2).

### Visualizing TLR gene expression using heatmaps

**Fig. 3:**
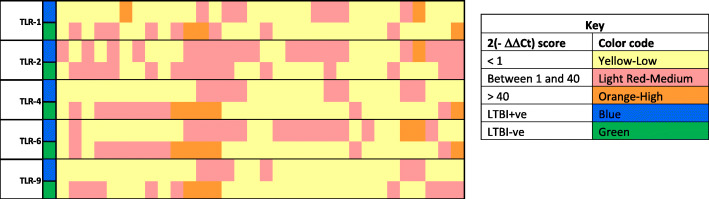
Heatmap of the TLR genes among latently infected and non-infected individuals; Horizontal axis indicates 2^(−ΔΔCt)^ values for TLR genes; score arbitrarily assigned taking 1 as a cut-off; colour codes indicate low, medium or high expression of the genes (Yellow indicates low, Light Red indicates medium and Orange indicates high expression); TLR = toll-like receptor

To better visualize the gene expression patterns among the study population, we did a heatmap using the ΔΔCt values of the TLR genes in LTBI and those non-infected (Fig. 3). This helps to clearly visualize the level of expressions of the genes in the study groups.

### Correlation between expression of TLRs

**Fig. 4:**
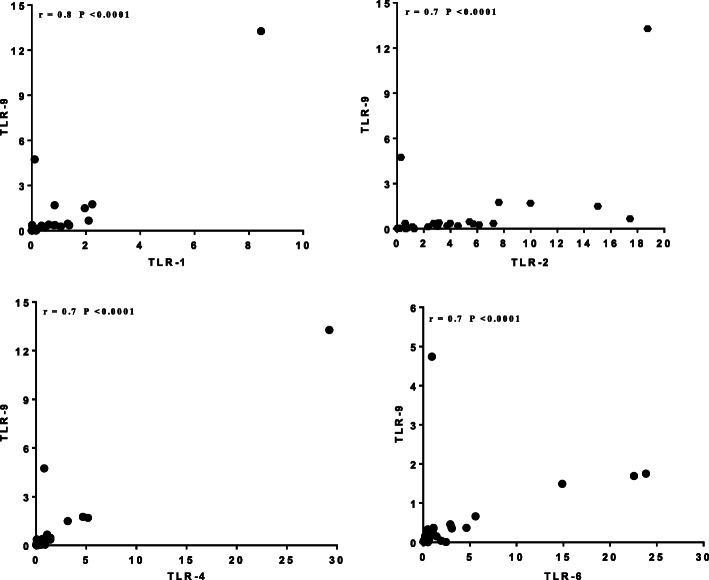
Correlation between TLR markers expressed on cell surface and endosomal regions; Spearman correlation coefficient (*r* = 0.70 and *P*-value < 0.0001); data were normalized relative to HuPO, the endogenous control; TLR = toll-like receptor

**Fig. 5:**
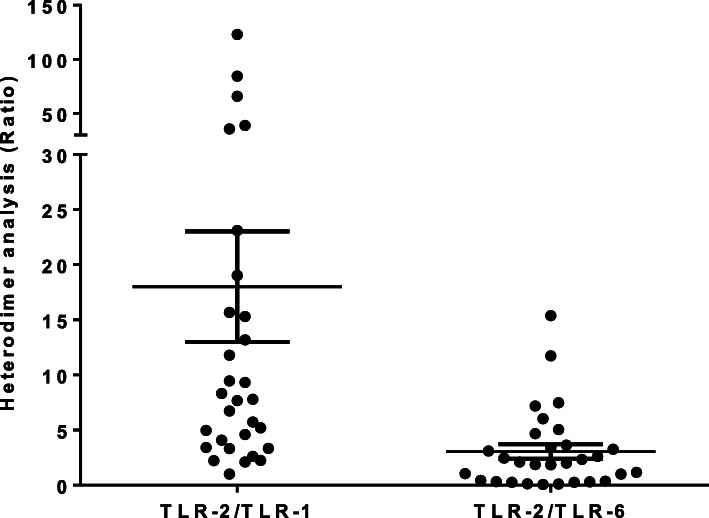
Heterodimer mRNA expression, shown as ratios in LTBI group relative to non-LTBI group; data normalized to HuPO; error bars indicate mean ± standard error of the mean (SEM); TLR = toll-like receptor

Similarly, correlation in the expression of surface TLRs with endogenously expressed markers was analysed and overall a positive correlation was observed between surface TLRs and an endogenously expressed TLR, TLR-9 (Fig. 4). A moderate correlation was also noted between the different surface TLRs (*r*^*2*^ = 0.6, *P*-value < 0.0001, data not shown). A similar comparison was done for those high and low expressed receptors to see possible correlations, especially those forming heterodimers; and the ratios (TLR-2/TLR-1 and TLR-2/TLR-6) were found to be higher (7.2 ± 1.12 (95% CI: 4.9, 9) and 3 ± 0.6 (95% CI: 1.6, 4.2)), among latently infected group when compared to non-infected groups, respectively (*P* = 0.004) (Fig. 5).

## Discussion

Early identification of individuals with LTBI, in particular those with a higher chance of progressing to a full blown active disease, is an important priority for TB control, especially in specific groups of population [18]. Currently there is no gold standard test for LTBI. Tools, currently existing to-date to fully control TB, have remained to be inefficient and biomarkers have been considered as alternatives options. Recent studies have also demonstrated efforts to show improved tools to diagnose LTBI over existing immunodiagnostic tools such as the interferon gamma release assays (IGRA); one to mention, a novel IGRA assay, the LIOFeron TB/LTBI assay, that has proven to show a higher accuracy to diagnose MTB infection/TB disease compared to QuantiFERON TB Gold Plus [19]. Nonetheless, despite extensive work to understand the role of adaptive immunity to *M. tuberculosis*, efforts to understand the role of innate immunity, particularly the fact that it can remember a previous exposure to TB via TLRs has become profound in recent years [20]. Toll-like receptors, one of the major innate immune markers, have been shown to be highly expressed during latent TB infection. These molecules are essential part of pattern-recognition receptor proteins expressed on the surface of the cell membrane or membrane of endocytic vesicles, having a role in the activation and triggering of pro-inflammatory cytokines, particularly in mycobacterial diseases [21]. The apparent increase in the relative expression of TLRs markers among latently infected individuals could be an indication for their role in the recognition of *Mtb* microbial components, such as lipoproteins, glycol-lipoprotein and lipomannan (LM) [22–25]. To mention few, the roles of TLR-1, TLR-2 and TLR-6 have been reported in the recognition of triacylated lipopeptides and diacylated lipopeptides of *Mtb*, TLR-4 in LPS recognition and TLR-9 homodimer in CPG DNA recognition [26]. In the present study, we observed an increased expression of TLR-2, TLR-4, TLR-6, and TLR-9 mRNA in latently infected group compared to non-infected, possibly indicating their role during latent TB infection through continuous stimulation and priming of immune system. Other studies have also reported a similar finding, although no significant difference in the expression of these markers was noted between active TB patients and latently infected individuals [27]. More studies have also indicated the occurrence of TLR stimulation that could result from interaction with circulating cytokines and modified by the cytokine milieu [27–29]. Although further confirmatory studies are warranted, our observation of enhanced expression possibly suggest a preliminary evidence for trained immunity, where MTB could potentially reside in and train the myeloid progenitor cells that will later be recognized by these receptors in the peripheral circulation. Studies have also demonstrated that MTB resides in hematopoietic stem cells, suggesting similar priming roles both in humans and animal models [30–34] and potentially leading to recognition of progenitors of the bacterial components by host TLRs in the blood circulation.

Toll-like receptor-2 often forms heterodimers with either TLR-1 or TLR-6, interacting with diacylated and triacylated lipoproteins, respectively [35, 36], where heterodimers recognize mycobacterial cell wall glycolipids like lipoarabinomanna (LAM), LM and mycobacterial glycoprotein [37]. To this effect, we compared ratios between TLR-2 and TLR-1 and TLR-6, where the ratios were significantly higher in latently-infected groups compared to those non-infected. The relative down regulation of TLR-1 gene in latently infected individuals could be related to the structural conformities required by TLR-1 to recognize microbial structures, as TLR-1 often needs TLR-2 to form a heterodimer to recognise microbial structures [26]. Similar observations were also reported by others, where the mRNA expression of TLR-1 was reduced in latently infected individuals [27].

Comparison of TLR expressions across age (between younger children and adolescents), however did not show any apparent difference. Since TLRs are mostly inborn immune receptors, expression of these markers across age may not be expected; however, exploring the expression of these genes across age may warrant further investigation with larger cohort to tease out immunological factors associated to higher incidences of TB infection among adolescents [13, 38–40] compared to younger children and further understand the significance of trained immunity.

Analysis of intra-compartment receptors (TLR-9) and surface receptors (TLR-1, TLR-2, TLR-4, and TLR-6), showed a strong positive correlation. Despite wide range of ligands recognized by TLRs, these receptors have been shown to share a common structural framework in their extracellular, ligand-binding domains, leading to cascaded pathways for pro-inflammatory cytokine signalling [24]. As a result, recognition of pathogen-associated patterns via TLRs direct the main pathways by which dendritic cells are activated and mature to provide signals to naive T cells and tailor specific immune responses [41]. Similar evidence demonstrated that TLR-2 heterodimer with TLR-1, TLR-6 and TLR-4 have been implicated as receptors involved in the recognition of mycobacterial antigens and activation of macrophages and dendritic cells [42]. On the other hand, synergic effects of polymorphisms between TLR-4 and TLR-9 has been shown to increase the risk of TB [25, 43]. In our study, a strong correlation was observed between expression of surface receptors and intracellular TLRs. Though each receptors can activate different components of the immune system, the synrgic effect and the activation of TLRs by their respective ligands has an informative role in directing acquired immunity [9, 10] and capable of modulating the adaptive immune response and differentiation of T cells [44, 45].

In conclusion, the present study demonstrated that upregulation of specific TLR markers, particularly TLR-2 and TLR-6 during latency, possibly suggesting the role these markers play during latent TB infection to maintain the continuous priming of the immune system and monocytes emigrating into LTBI site in the lung and better able to sustain local immunity. Likewise, a strong correlation observed between endosomal receptors (like TLR-9) and surface TLRs during latency may show their role in maintaining the steady interaction between the dormant TB bacilli and host immunity. One could possibly argue an association between two variables (such as in this case between LTBI and TLR expression) in a cross-sectional study can simply imply cause-effect, while each variable could be casually related to a third or different underlying factor(s). Given that inflammatory biomarkers are normally distributed in a population, some individuals may always have higher biomarker levels than others, due to a number of usually unexplained reasons, some of which could also be associated with trained immunity or otherwise. It is also possible that associated underlying heightened immune mechanisms could simply contribute to increased ease to detect LTBI in one of two populations even though the true prevalence of latent TB in each population could be identical. Therefore, to further validate such observations designing studies in controlled groups such as before and after isoniazid therapy and also exploring the priming effect of MTB in milieu of myeloid progenitor cells is strongly warranted. In addition, exploring these and additional innate immune markers in a wider cohort of participants that also includes TB patients is warranted to elucidate whether these genes can be used as possible biomarkers in future booster TB vaccine trials.

## Supplementary Information

**Additional file 1.**

## Data Availability

The datasets generated and/or analysed during the current study are still under use for an on-going study and will be available from the corresponding author on reasonable request.
