# Supplementary material for: Ex vivo mRNA expression of toll-like receptors during latent tuberculosis infection
Source: BMC Immunol. 2021 Jan 28;22:9. doi: 10.1186/s12865-021-00400-4 (PMC7842038; doi:10.1186/s12865-021-00400-4)

*Ex vivo* mRNA expression of toll-like receptors during latent tuberculosis infection

Birhan Alemnew^1,2^, Soren T. Hoff^3^, Tamrat Abebe^4^, Markos Abebe^2^, Abraham Aseffa^2^, Rawleigh Howe^2^, Liya Wassie^2*^

# Supplementary data

# Graphical presentation of mRNA expression profile of housekeeping gene (HuPO), non-template control (NTC) and target genes (TLRs) using *qRT-PCR* from representative experiments; all pictures are shown in log scale; threshold cycles were arbitrarily set considering a minimum CT value difference between duplicate samples in each experiment.


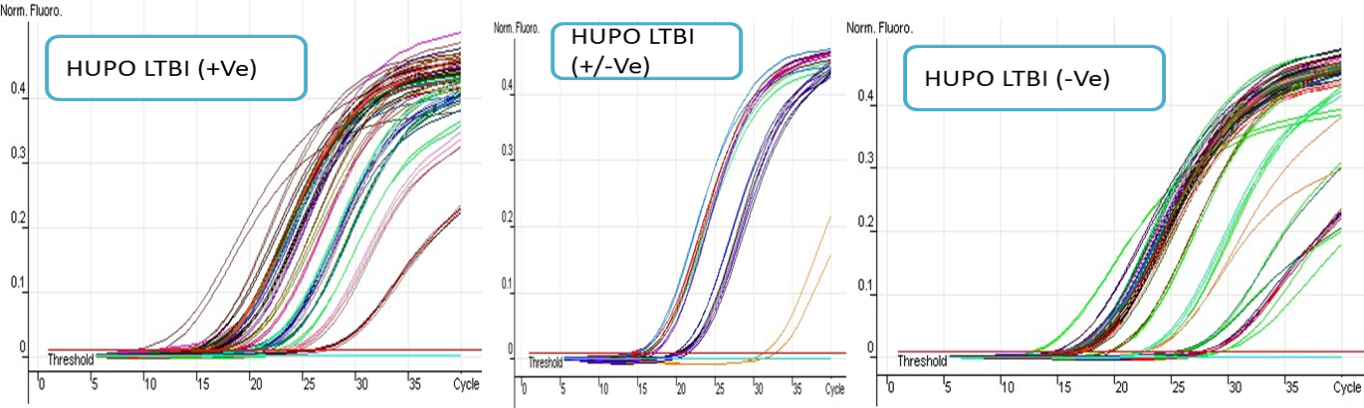


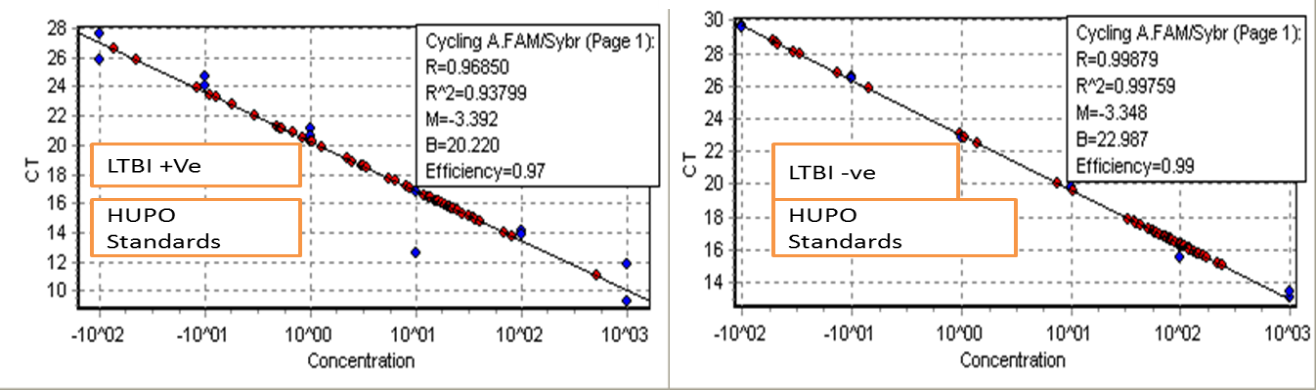


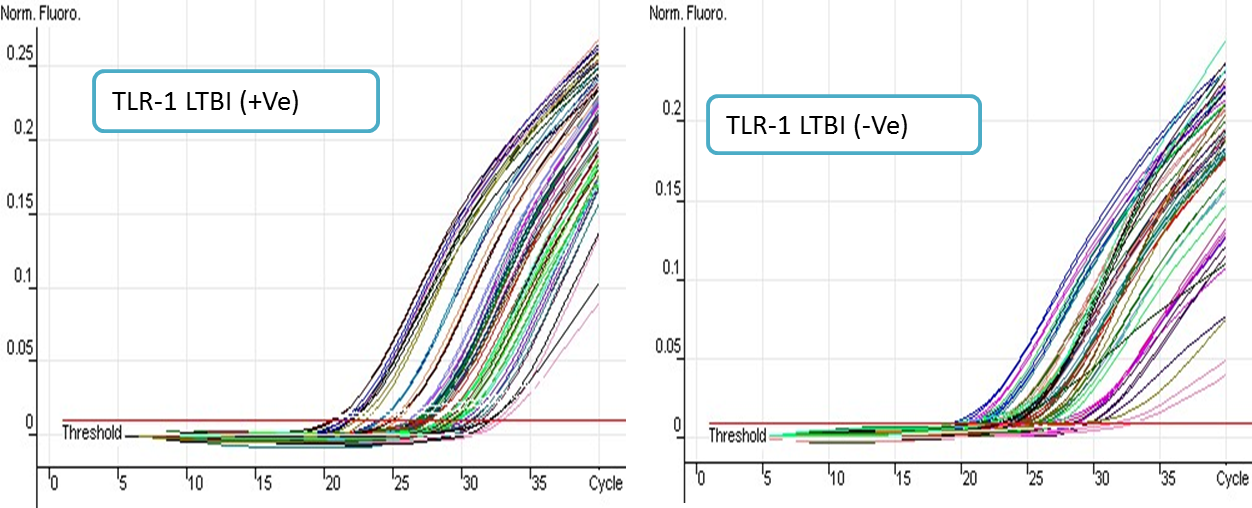


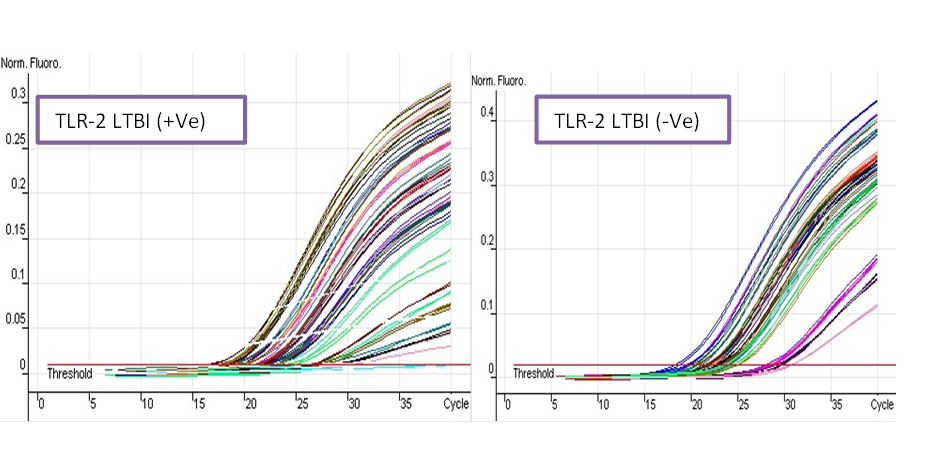


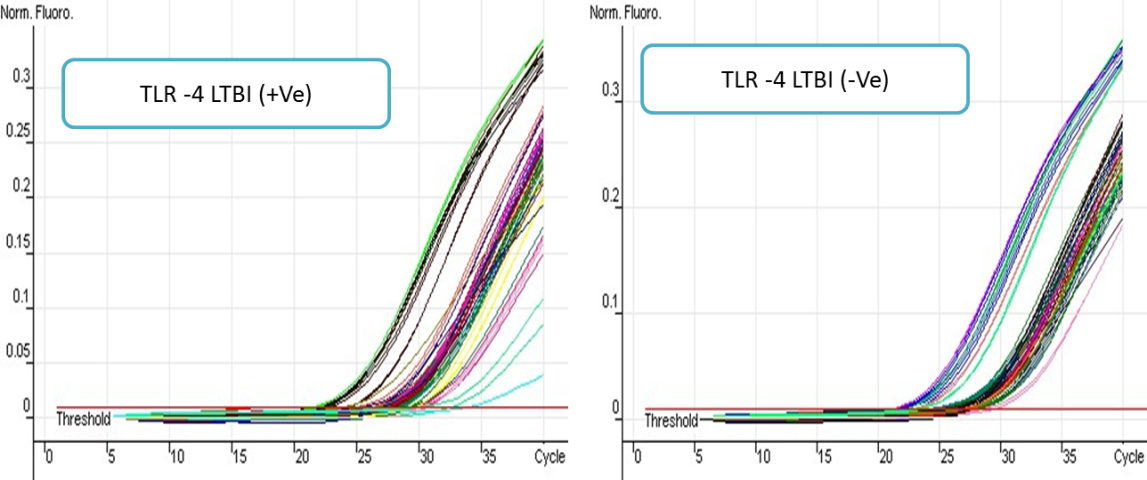


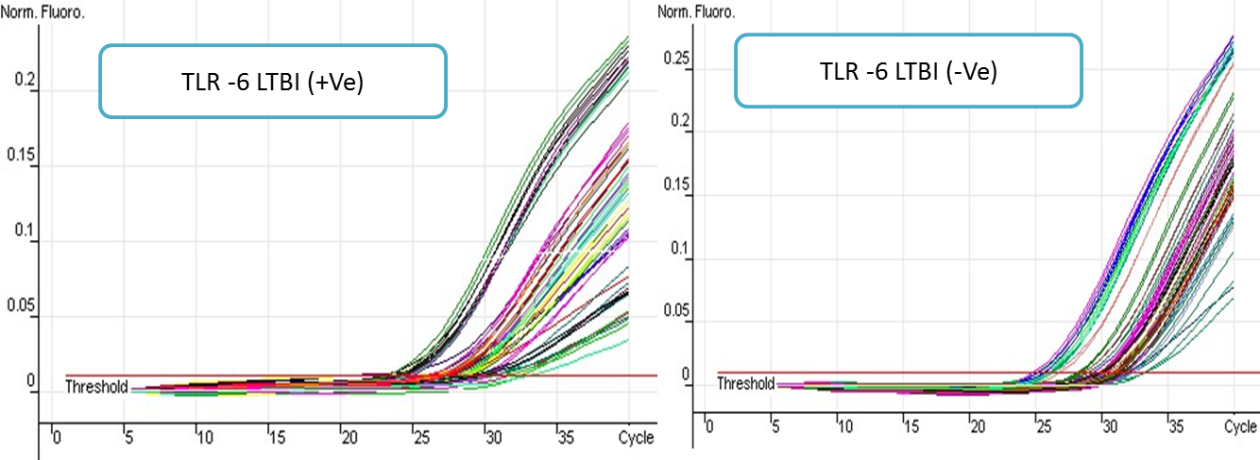


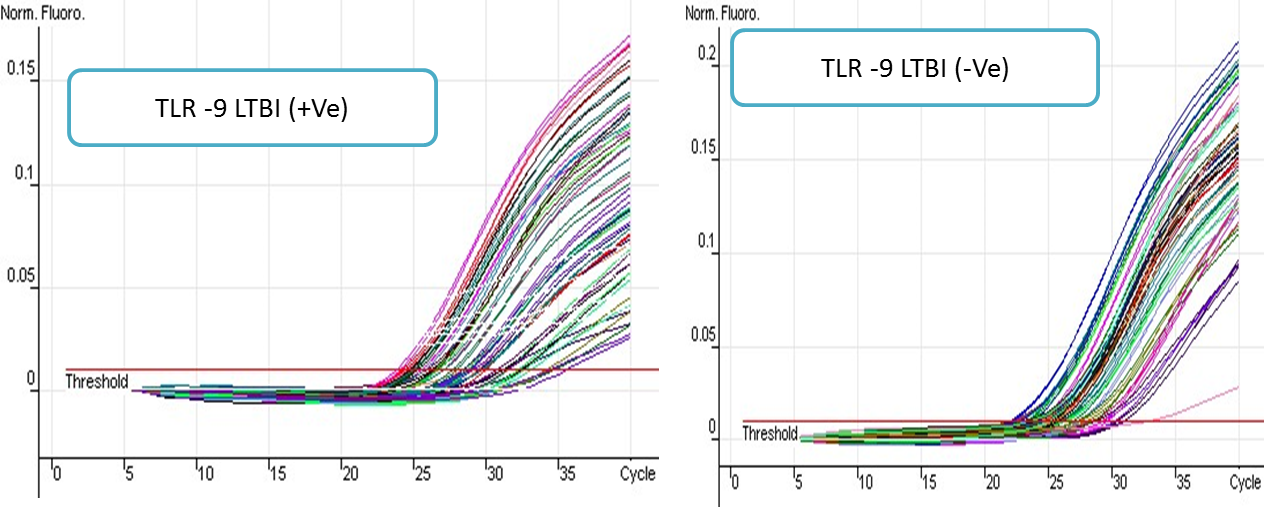

Supplement: Supplementary file 1 — Additional file 1. [file 12865_2021_400_MOESM1_ESM.doc]
